# Plazomicin against Multidrug-Resistant Bacteria: A Scoping Review

**DOI:** 10.3390/life12121949

**Published:** 2022-11-22

**Authors:** Aniello Alfieri, Sveva Di Franco, Valerio Donatiello, Vincenzo Maffei, Ciro Fittipaldi, Marco Fiore, Francesco Coppolino, Pasquale Sansone, Maria Caterina Pace, Maria Beatrice Passavanti

**Affiliations:** 1 Department of Elective Surgery, Postoperative Intensive Care Unit and Hyperbaric Oxygen Therapy, A.O.R.N. Antonio Cardarelli, Viale Antonio Cardarelli 9, 80131 Naples, Italy; 2 Department of Women, Child and General and Specialized Surgery, University of Campania Luigi Vanvitelli, Piazza Miraglia 2, 80138 Naples, Italy; 3 Unit of Critical Care, Hospital “Ospedale Pellegrini”, Via Portamedina alla Pignasecca 41, 80134 Naples, Italy

**Keywords:** antimicrobial resistance, bloodstream infections, multi-resistant *Enterobacterales*, pyelonephritis, plazomicin, scoping review, urinary infections

## Abstract

Plazomicin is a next-generation semisynthetic aminoglycoside antibiotic that can be used to treat infections by multi-resistant bacteria. It is effective against many bacteria-producing carbapenemases or other specific hydrolases. This scoping review aims to define the role acquired by plazomicin from its approval by the FDA (US Food and Drug Administration) in 2018 to the present day. Furthermore, we aim to provide a base for a future meta-analysis. This project was conducted following the recommendations presented in the PRISMA extension for scoping reviews and the JBI Manual for Evidence Synthesis. Among 901 potentially engaging citations, 345 duplicates were removed, and only 81 articles were selected for the analysis. According to the data analysis, plazomicin has been used to treat urinary tract infections, bloodstream infections, and ventilation-associated pneumonia. The pathogens killed included multi-resistant *E. coli*, *K. pneumoniae*, *A. baumannii*, *P. aeruginosa*, and *S. aureus*. Plazomicin can be a manageable, valid non-beta-lactam alternative for treating multi-resistant bacteria infections.

## 1. Introduction

Plazomicin (formerly ACHN-490; (C25H48N6O10)) is a new-generation antibiotic approved in June 2018 by the US FDA (United States Food and Drug Administration) that is active on numerous pathogens conventionally resistant to classic aminoglycosides [1].

Aminoglycosides are a class of broad-spectrum antibiotics discovered for the first time in 1944. Streptomycin was the first aminoglycoside to be discovered. Aminoglycosides are bactericidal drugs, and their actions on the target site include ribosomal blockade, misreading in translation, membrane damage, and irreversible uptake of the antibiotic.

Aminoglycosides have the chemical structure of a polycationic sugar with amino and hydroxyl domains that characterize the pharmacokinetic and pharmacodynamic profiles. Their polycationic structure gives them the ability to bind nucleic acids, showing a strong affinity for the rRNA of both eukaryotes and prokaryotes. Quite a few studies, summarized in the mini-review by Kotra and colleagues, have focused on aminoglycosides’ targets (the specific binding sites of aminoglycosides to rRNA).

It is interesting that resistance to aminoglycosides is not due to mutations of the binding sites such as the rRNA of the target pathogen; in fact, rRNA is always well protected as it is essential for protein synthesis and the survival of the bacteria. In addition, rRNA is encoded by numerous copies of genes, so for the target pathogens, developing resistance mechanisms that involve mutations in rRNA would be harmful and very complex, requiring modifications of numerous gene sequences [2].

However, the development of antibiotic resistance mechanisms due to RNA mutations is not to be excluded.

These resistance mechanisms have been found after the administration of sodarin (antimycotic) and erythromycin. In particular, in the case of sodarin administration, the resistance was due to mutations of the ribosomal protein L10e [3], and in the case of erythromycin administration, the resistance was due to mutations of the L4/L22 protein [4].

These cases of resistance caused by the mutation of ribosomal proteins are still exceptional cases, and similar cases of mutations have not yet been found for aminoglycoside resistance.

Focusing our attention on the mechanisms of resistance to aminoglycosides, the most frequent finding was the production of inactivating enzymes. For years, *Enterobacterales* had been the undisputed target of aminoglycosides, but unfortunately, these bacteria developed the ability to produce inactivating enzymes such as aminoglycoside-inactivating enzymes (AMEs—aminoglycoside-modifying enzymes). The production of AMEs and the unfavorable toxicity/benefit ratio in patients suffering from infections caused by AME-producing bacteria have limited old aminoglycosides’ use [5]. Based on these considerations, the necessity of a new antibiotic with undisputed efficacy and limited toxicity, such as plazomicin, is evident.

Plazomicin, being a semisynthetic product, has a unique structure that is resistant to inactivation by numerous enzymes produced by multi-resistant bacteria. Table 1 describes the domains present or absent in plazomicin when compared with traditional aminoglycosides and the ability to resist enzymatic inactivation [6].

Table 1 defines the association between the molecular structure of plazomicin and its ability to resist inactivation by AMEs in comparison with old aminoglycosides.

The chemical characteristics of plazomicin differ from those of classical aminoglycosides due to the lack of hydroxyl groups typical of amikacin, gentamicin, and tobramycin. These hydroxyl groups are the cause of the susceptibility of amikacin, gentamicin, and tobramycin to the inactivating enzymes. 

Furthermore, plazomicin has an unsaturated hydroxyethyl group and a substitution with 4-amino-2-hydroxybutanoic acid which protects it from the attack of the numerous hydrolytic enzymes described in Table 1.

With these chemical characteristics, it is prescribable to treat infections caused by *Enterobacterales* resistant to classical aminoglycosides, but it is also effective even in the case of bacteria resistant to carbapenems (producers of carbapenemases such as IMP, VIM, and NDM) and to colistin (polymyxin B) and ESBL-producing bacteria [7]. 

To date, plazomicin has been used to treat infections in ICUs affecting the urinary tract, including pyelonephritis, bloodstream infections, and ventilator-associated pneumonia (VAP) [8].

Like all aminoglycosides, it is not very effective in an anaerobic environment as its transport within the cell’s cytoplasm is energy-dependent and influenced by pH. Therefore, in the case of an acidic environment (i.e., acidic urine), cell penetration is reduced [9].

However, plazomicin lacks activity against bacteria encoding genes for 16S rRNA methyltransferases (isolated mainly in East Asia and, unfortunately, sometimes co-expressed with NDM) [10]. 

The synergy with other antibiotics used for the treatment of complicated infections is also interesting [11].

It should be considered that, like all aminoglycosides, it is a drug with linear, first-order elimination; low protein binding (~20%); and predominantly renal excretion (glomerular filtration without undergoing hepatic or plasma metabolism). According to the safety profiles of traditional aminoglycosides, they have always shown numerous side effects, especially when compared to beta-lactams. However, considering their great efficacy, despite the several side effects, aminoglycosides’ clinical use has not been reduced.

However, while administering aminoglycosides, monitoring of the two main types of toxicity should be kept in mind, namely ototoxicity and nephrotoxicity. A dose reduction has to be considered in cases of patients with impaired renal function [12].

On the one hand, nephrotoxicity is a reversible type of damage due to the accumulation of drugs in the cortical area of the kidney. The accumulation mechanism foresees that the aminoglycosides are sequestered by the epithelial cells of the proximal tubules following their plasma excretion and glomerular filtering. It is a reversible process that depends on the regenerative capacity of the tubular epithelial cells [13].

Ototoxicity, on the other hand, constitutes direct oxidative damage to the vestibular organ, to the cochlea and its hairy cells, and to the respective cranial nerves. This damage is irreversible and permanent, and for this reason, it must be prevented. The use of antioxidant molecules such as N-methyl-D-aspartate (NMDA) receptor antagonists has been found to be useful in reducing ototoxicity if administered simultaneously with aminoglycosides. Among the drugs that have been tested in combination with aminoglycosides and found to prevent ototoxicity, there are both easily prescribed drugs such as salicylate, aspirin, N-acetylcysteine, dexamethasone, mitoquinone, and melatonin as well as drugs such as tacrolimus and SkQR1 which are for hospital or experimental use only [14].

Given that all these drugs, although administered in combination, reduce but do not cancel the ototoxicity of aminoglycosides, it is important to note that plazomicin is non-ototoxic. Therefore, its administration has been shown to be safe as well as effective and practical [15].

The manageability of plazomicin, above all, is given by the method of administration. The method of administration approved by the US FDA is a single intravenous injection at a dose of 15 mg/kg, infused over 30 min per day [16].

Currently, there are not many literary reviews on this topic, and therefore, we believe a scoping review could be interesting to describe and provide an overview of the current data presented in the literature on this area. This scoping review aims to be a preliminary step to a systematic review on this topic. We aim to describe in a clear and transparent way the current state of the literature regarding the clinical and in vitro use of plazomicin to treat infections from pathogens resistant to conventional antibiotics.

## 2. Materials and Methods 

This review was conducted following the methodology proposed in the PRISMA extension for scoping reviews (PRISMA-ScR) [17] and the recommendations suggested in the JBI Manual for Evidence Synthesis [18].

Our project aim was to conduct a scoping review, dividing the selection and analysis work in five phases. The five phases included a first phase of formulation of the clinical question (1), a second phase for the definition of the search strategy (2), a third phase of identification of the relevant studies (3), a fourth phase of relevant studies’ selection (4), and a final phase of data synthesis and result presentation (5).

### 2.1. Clinical Question

The question underlying this scoping review was as follows: “What is the current clinical use of plazomicin in severe infections with multidrug-resistant pathogens?”. The formulation of the clinical question was conducted by applying the PCC methodology (population/concept/context) [18] and schematized as follows: population—severely infected patients; concept—effectiveness and safety of plazomicin; context—infection from multi-resistant bacteria.

### 2.2. Research Strategy and Data Sources

For the planning of the search strategy, an initial survey was conducted to identify keywords and mesh terms to be used. The databases consulted were MEDLINE (via Ovid), EMBASE, Cochrane Library, and the ClinicalTrial.gov and EU-CTR registries. The terms used were “plazomicin” and “ACHN-490”, with strings specifically built for each database (detailed search strings are available in Appendix A). No restrictions on publication date were applied.

### 2.3. Citation Management

All the studies thus selected were imported into a bibliographic manager software (Endnote v. 20) [19] with which the duplicate citations were removed; other duplicates were identified in the subsequent phases and manually removed. The list obtained was used to build a table on an electronic spreadsheet (Microsoft Excel, v. 2209) [20] with information about year of publication, authors, title, abstract, and DOI. This table was used in the subsequent stages of screening and data synthesis.

### 2.4. Inclusion Criteria

Only clinical studies (randomized controlled trials, cohort studies, and observational studies), published in English with no restrictions on publication date, were included. In vitro studies on isolates were included in the analysis separately. Systematic reviews were analyzed to detect the presence of further papers not included in previous searches.

### 2.5. Title and Abstract Screening

In the first screening phase, two authors (A.A. and S.D.F.) independently assessed the list of works obtained in the previous phases through title and abstract analysis, excluding the works considered irrelevant to the clinical question according to the established inclusion criteria. Disagreements between the authors were resolved through discussion of each discordant case or by consulting a third reviewer (V.D.).

### 2.6. Quality Assessment and Extrapolation of the Characteristics of the Studies

In this phase, papers were excluded if they were not considered eligible by the inclusion criteria. The studies considered relevant after the first screening phase were analyzed in full-text, by two authors (A.A. and S.D.F.) independently, to assess study quality. The quality of each study was assessed using Rob2 (Version 2 of the Cochrane risk-of-bias tool for randomized trials) or ROBINS-I (Risk Of Bias In Non-Randomized Studies—of Interventions). Subsequently, each study of the selected ones was consulted by two authors (A.A. and S.D.F.) independently to extrapolate data relating to the type of study and the clinical setting. For each of the clinical studies (RCTs, cohort studies, or observational studies), the characteristics of the outcome were extrapolated. 

### 2.7. Data Synthesis

The data collected in the previous phase were used to create a datasheet (Microsoft Excel, v. 2209) [20]. The data were then imported into the R environment (RStudio, v. 2022) [21] and analyzed. The results were discussed through summary and descriptive statistics. The presentation was simplified using devices such as graphs and tables.

## 3. Results 

### 3.1. Identification of the Eligible Studies

The search strategy led to the recognition of 901 potentially engageable citations in this scoping review. After automatic removal of the duplicates (*n* = 345), evaluation of the abstracts and titles of the remaining 556 papers was started. After applying the inclusion criteria, we excluded 226 review studies, and 147 papers, even if present in the list to be screened, were off-topic. Letters to the editor (*n* = 20), academic papers on plazomicin pharmacology (*n* = 14), congress expositive papers (*n* = 5), studies on animal models (*n* = 5), academic papers on genome and plazomicin (*n* = 5), and two guidelines were excluded. In this phase of the analysis, further duplicates (*n* = 27) were discovered and removed manually by the authors. Considering the sum of the duplicates and the ineligible studies, the total number of excluded papers in this phase was 451.

Next, the full texts of 108 papers were examined. Of these, 3 were off-topic and 10 were systematic reviews; the remaining 96 studies were included in the analysis.

A list of the exclusion criteria of the identified articles during the screening phase is presented in Table 2.

Among the 96 studies included according to the quality assessment process, all were considered as being of good quality and eligible. 

**Figure 1:**
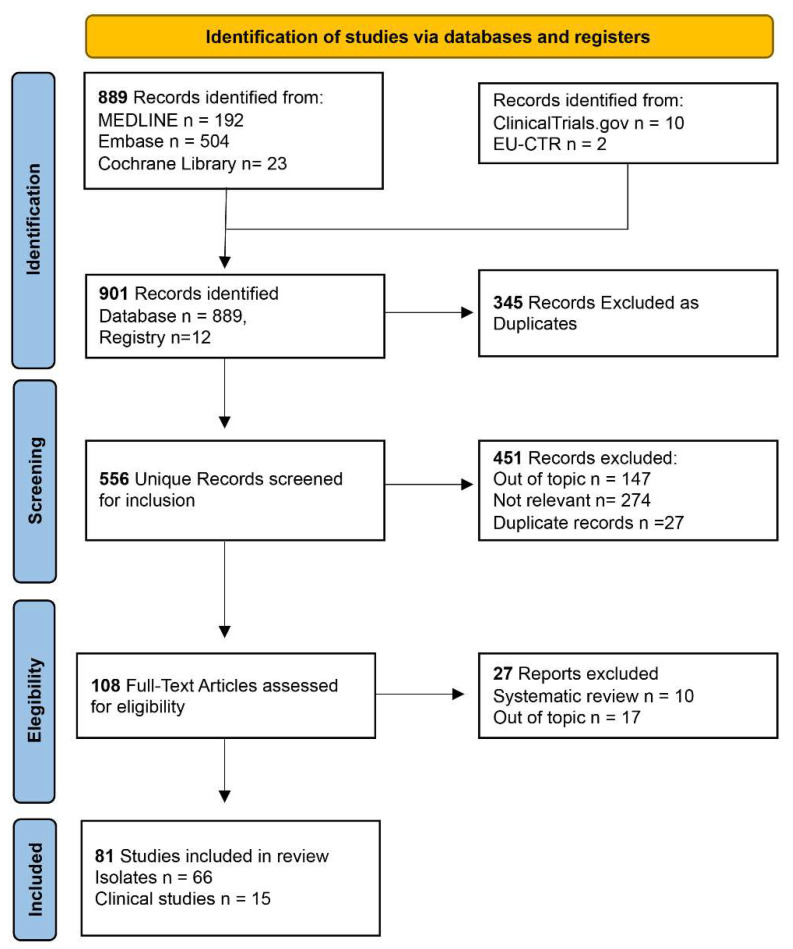
PRISMA flowchart describing the fourth phases that led to the selection of the 81studies included in the scoping review.

This process has been schematized in the PRISMA flowchart [17] presented in Figure 1.

### 3.2. Characteristics of the Included Studies

**Figure 2:**
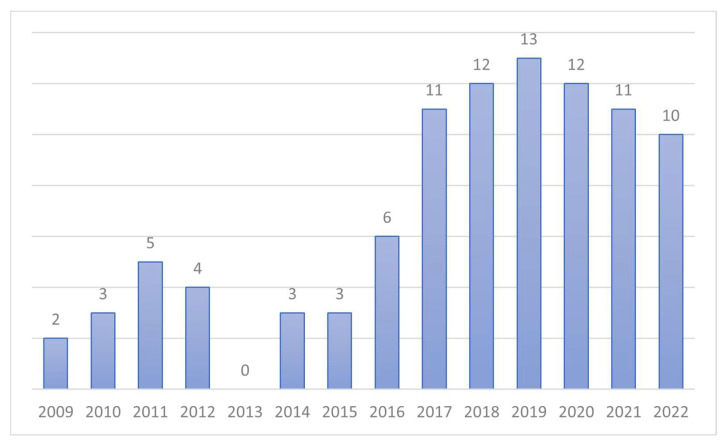
Visual description of the included articles divided per year of publication.

All the selected studies were published between 2009 and 2022, with more than 70% of the works published after 2017. Figure 2 is a visual description of the number of studies per year among the included ones.

It should be noted that the first works on this topic were published in 2009, with an increasing rate of publication due to the rise in popularity of plazomicin in 2017. Nearing the date of approval by the US FDA (2018) [22], there was a peak of interest in the field.

In total, 66 papers concerned the spectrum of activity of plazomicin on clinical isolates. There were 20 detected clinical studies, of which 15 were RCTs and 5 were observational studies or case reports.

Among the included clinical studies, 40% (*n* = 6) of the RCTs were phase 3 studies, 46.66% (*n* = 7) were phase 1 safety studies, and 13% (*n* = 2) were phase 2 studies. Twelve RCTs from registries of clinical trials were identified (10 studies from www.clinicaltrial.gov and 2 studies from the European Union Clinical Trials Register (EU-CTR)). 

Regarding the clinical setting in which plazomicin was used, including all the phase 2 and 3 studies, in nine cases, it was administered for patients with urinary tract infections (UTIs); in seven studies, it was used for bloodstream infections (BSIs); in two studies, plazomicin was the elective drug for both BSI and UTI; and in one study, it was employed to treat ventilation-associated pneumonia (VAP) and BSI. 

Focusing the attention on phase 1 studies, we identified seven studies conducted on healthy volunteers and three studies where plazomicin was administered to patients with chronic renal failure. The three case reports concerned urosepsis from multidrug-resistant germs treated with plazomicin.

Table 3 offers a synthesis of the RCTs published on the topic. 

The included clinical trials show plazomicin action compared with other antibiotics currently employed in the fight against multidrug-resistant bacteria. Table 4 reports a list of the comparator antibiotics used in the clinical trials detected and included in this scoping review. 

Looking through the data collected on clinical isolates, there were 66 studies detected on clinical isolates tested with plazomicin. The data obtained from isolates were derived mainly from the USA (United States of America) (54.55%), and only 21.21% were from European treatment centers. These studies on isolates tested with plazomicin provide a description of the bacteria isolated and treated, and they assessed the activity of plazomicin on various pathogens, as shown in Table 5.

The pathogens against which the activity was tested were in almost half of the cases (46.97%) bacteria belonging to the order of *Enterobacterales*. Additionally, 24.24% of the studies (16 papers) analyzed the plazomicin resistance spectrum specifically for *Klebsiella Pneumoniae*. Other isolated pathogens were *Escherichia Coli* (9.09%), *Staphylococcus aureus*, *Pseudomonas Aeruginosa*, and *Acinetobacter baumannii* (4.55%). All pathogens of the ESKAPE group are present in the list. 

In 53 studies, the activity of plazomicin on strains of antibiotic-resistant pathogens was analyzed, as synthesized in Table 6. 

The most frequent strains were carbapenem-resistant *Enterobacteriaceae* (CRE, 23/53), multidrug-resistant bacteria (MDR, 15/53), and carbapenemase-producing *Klebsiella pneumoniae* (KPC 6/53). Other less represented strains were extended-spectrum beta-lactamase *Escherichia coli* (ESBL-E), methicillin-resistant *Staphylococcus aureus* (MRSA), aminoglycoside-modifying enzymes (AMEs) productors, and New Delhi metallo-beta-lactamase (NDM) productors. 

For the in vitro spectrum of action, 66 studies on isolates defined how plazomicin is effective against a wide range of pathogens and on different strains of multi-resistant bacteria with good results. A systematic review of the effects would help quantify the clinical response of plazomicin; however, more randomized trials with larger populations are needed.

According to the results of this scoping review, plazomicin has been used in clinical practice to treat infections due to multidrug-resistant bacteria, especially infections of the urinary tract, bloodstream infections, and pneumonia related to mechanical ventilation. There is good evidence of its activity in treating complicated urinary tract infections; thus, other clinical settings should be further studied. 

At the moment, the data of the 15 clinical trials in which the drug was administered make it clear how effective it is already in monotherapy.

## 4. Discussion 

Infections due to multidrug-resistant pathogens have become more and more frequent and are widespread all over the world [23]. 

Worldwide, the highest risks of mortality and morbidity have been reported for infections caused by multidrug-resistant Gram-negative bacteria. 

Among the bacteria that demonstrated a relevant ability to develop resistance, we found ESBL-*Enterobacterales* and carbapenemase-producing *A. baumannii*, *P. aeruginosa*, and *Enterobacterales*. The treatment of these infections is becoming increasingly complex in terms of both initial empirical therapy and treatment after microbiological confirmation. Hence, it is essential that antibiotics, especially broad-spectrum and new-generation ones, are used sparingly, even in this era where the COVID-19 pandemic seems to have promoted the abuse of antimicrobial treatments not often justified by pathophysiology [24].

It is important not to lose the path given by antimicrobial stewardship and not to forget how antibiotics such as beta-lactams, once the mainstay of the treatment of infections, are now frequently vulnerable to inactivation by beta-lactamase—the same fate to which we condemned other classes of antibiotics that were once very effective, such as carbapenems now inactivated by carbapenemases. The particular concern with carbapenem resistance mechanisms is related to the recent appearance in Gram-negative bacteria of five carbapenemases’ genes all mediated by plasmids and therefore having a high horizontal transmission rate. These are IMP, OXA-48-type, NDM, KPC, and VIM.

The various resistance mechanisms developed by multidrug-resistant bacteria against broad-spectrum and new-generation antibiotics are rapidly increasing, leading to a reduction in our treatment options for the bone [25].

Patients suffering from pathologies caused by multidrug-resistant bacteria and the healthcare professionals that have the responsibility to treat these complex infections face an uphill clinical therapeutic path with an often poor prognosis [26,27].

However, we still have some next-generation drugs that can help us treat some of the worst healthcare-related infections when used correctly and sparingly. Among these next-generation drugs is plazomicin, the subject of this scoping review [28]. 

The use of plazomicin could be a valid strategy in treating infections caused by pathogens resistant to carbapenems, especially in countries where the rate of resistance to these antibiotics is increasing [29,30]. 

However, considering that an efficient weapon against multidrug-resistant bacteria should be used carefully, we decided to propose a scoping review to identify the operative setting in which plazomicin is widely used. 

Plazomicin is used in three main settings. The phase 3 studies presented in the results describe plazomicin as a monotherapy or in association with another antimicrobial to treat complicated UTI (cUTI), BSI, and VAP. The data on the use of plazomicin seem to be promising, but a data meta-analysis is desirable to clearly define the advantages. 

Two indications have been clearly identified from a general overview of the data for plazomicin: cUTI in a phase 2 trial and the EPIC trial [31], and serious CRE infections (including BSI, hospital-acquired pneumonia (HAP), and VAP) in the CARE trial [32]. 

In the EPIC study, plazomicin was found to be non-inferior to all protocol-established endpoints when compared to therapeutic standards for cUTI. However, it has shown excellent bactericidal action against numerous bacteria that express resistance to aminoglycosides, beta-lactams, fluoroquinolones, and carbapenems. In any case, despite the positive data from the CARE study, due to the small number of patients treated, the FDA has not approved the use of plazomicin in the case of infections by carbapenemase-producing bacteria. Furthermore, data on adverse events seemed to asseverate that plazomicin-related adverse events were more debilitating compared with colistin-related ones. 

Thus, the FDA approved plazomicin with a black box warning for aminoglycoside class effects (nephrotoxicity, ototoxicity, neuromuscular blockade, and pregnancy risk) [22].

The side effects placed on the scales and the benefits derived from antibiotic therapy with plazomicin emphasize the drug’s safety compared to traditional aminoglycosides.

As presented in this review, there have been seven phase 1 studies, three of them reporting the drug’s general safety profile and general pharmacology on healthy volunteers and showing few side effects and an effective concentration of antibiotic in the apparatuses considered as the effector. One study conducted to evaluate the presence of an interaction with metformin showed no notable alterations in the blood concentration of metformin with simultaneous intake of plazomicin [33]. Two studies on patients with impaired renal function reported good tolerance of the drug even in patients with advanced kidney disease or on dialysis. 

Since plazomicin belongs to the class of aminoglycoside antibiotics, marked renal toxicity would be expected. However, the renal toxicity showed by plazomicin is not dissimilar to that caused by meropenem (3% of patients treated with plazomicin showed renal function impairment). It must be considered that renal damage caused by plazomicin is reversible. Most patients (about 80%) already at the discharge visit after treatment showed complete renal function [31].

Additionally, one study evaluating the cardiac effect of plazomicin administration showed no significant alterations in the QT length [34].

In the EPIC trial, which enrolled the most patients out of any other trial (303 received plazomicin), the most common adverse events reported were decreased renal function (3.7%), diarrhea (2.3%), hypertension (2.3%), headache (1.3%), nausea (1.3%), vomiting (1.3%), and hypotension (1.0%) [31]. 

Overall, serious adverse events (ototoxicity) reported in trials were documented in less than 2% of patients. Keep in mind that the context of clinical trials is different from the context of clinical practice. Therefore, caution should always be exercised in patients with previous kidney or inner ear diseases. Furthermore, since traditional aminoglycosides are associated with other antibiotics, the association between plazomicin and other antibiotics (i.e., meropenem, levofloxacin, ceftazidime-avibactam, meropenem-vaborbactam, cefiderocol, ceftolozane-tazobactam, and tigecycline) has also been evaluated [11]. 

Already in monotherapy, it should be remembered that plazomicin improves the prognosis of patients suffering from multidrug-resistant bacteria and reduces the risk of degeneration of the infection into septic shock by reducing hospitalization times [35]. 

Plazomicin association with these antibiotics seems to prevent the development of antibiotic resistance without risks [36].

On the topic of resistance development to plazomicin, pathogens demonstrating resistance to plazomicin were rarely encountered across these clinical trials; only six isolates cultured in the CARE trial (two from the plazomicin arm and four from the colistin arm) and seven isolates in the EPIC trial had baseline MICs resistant to plazomicin. In the EPIC trial, only one of the patients required additional antimicrobial therapy following the initial administration of the study drug. All these isolates were confirmed to express 16S rRNA methyltransferases. Caution should be taken when using plazomicin to treat NDM-producing Enterobacteriaceae. NDM-producing phenotypes associated with the expression of 16S rRNA methyltransferases have been sporadically noted in the US; however, other countries in Europe and Asia have documented a more endemic prevalence. In 22% of the cases, there was a co-expression of OXA-48-like carbapenemases too. It is auspicial, before plazomicin administration, to use commercially available rapid detection tests that are able to identify the presence of carbapenemases, including NDM. Clinicians are advised, as always, to consult their local antibiograms and subsequently recommend empiric therapy and de-escalate the antibiotic dosage appropriately as new patient data are made available.

Although plazomicin is a reasonably expensive drug to be used parsimoniously according to what discussed above, it allows for saving on the costs of the entire course of care for patients suffering from serious infections such as complicated urinary tract infections, bloodstream infections, and nosocomial pneumonia not sensitive to drugs such as carbapenems [37]. 

## 5. Conclusions

Plazomicin is a new-generation semisynthetic aminoglycoside with activity against MDR *Enterobacterales*. It has demonstrated non-inferiority compared to meropenem for the treatment of cUTIs. Furthermore, plazomicin may be a good option to treat cUTIs caused by pathogens with poor sensitivity to carbapenems.

Plazomicin is administered once daily, if not less frequently based on underlying renal function, and has a short 30-minute administration duration, resulting in a convenient treatment option for the outpatient antibiotic treatment (OPAT) setting. 

Adverse events are generally low in frequency and similar to other available classical aminoglycosides and include nephrotoxicity, ototoxicity, headache, nausea, vomiting, diarrhea, hypertension, and hypotension.

According to what has been reported in this scoping review, plazomicin may be a golden solution to deal with the most complex infections due to multidrug-resistant bacteria, but there is a gap in the literature that is defined by the lack of data from clinical trials using plazomicin to manage BSIs and VAP compared with plazomicin use for UTI control. It seems to be a powerful, practical, and safe tool for the control and resolution of infections that would not respond with favorable outcomes to the use of classic broad-spectrum antibiotics, but this new antimicrobial drug, to be properly exploited and have its effectiveness maintained over time, should be administered when necessary and sparingly.

In conclusion, plazomicin can be considered a promising non-β-lactam alternative for the treatment of infections caused by MDR *Enterobacterales*, and a meta-analysis on this topic is advisable to help us clarify its clinical and therapeutic role.

## Figures and Tables

**Table 1:** Synthesis of the molecular characteristics that give plazomicin resistance to AMEs.

Plazomicin Molecular Characteristics	Protection against AMEs Conferred from the Molecular Characteristics	Traditional Aminoglycosides Inactivated by AMEs
Lacks hydroxyl groups in the 3′ and 4′ positions	O-nucleotidyltransferase (ANT(4′))	Amikacin and tobramycin
	O-phosphotransferase (APH(3′))	Amikacin
Presence of unsaturated hydroxyethyl group at position 6′	N-acetyltransferase (AAC(6′))	Amikacin, gentamicin and tobramycin
N-1 substitution with 4-amino-2-hydroxybutanoic acid	N-acetyltransferase (AAC(3′))	Gentamicin and tobramycin
	O-nucleotidyltransferase (ANT(2′))	Gentamicin and tobramycin
	O-phosphotransferase (APH(2′))	Amikacin, gentamicin, and tobramycin

**Table 2:** Exclusion criteria in screening phase.

Review	226
Off-topic	147
Letter	20
Pharmacology	14
Congress	5
Animal	5
Genome	5
Guideline	2
Duplicates	27
Total Excluded	451

Schedule of the number of excluded articles during the screening phase, divided according to the exclusion criteria.

**Table 3:** Report of published RCTs on plazomicin.

Year of Publication	Identification Number	Study Phase	Study Title	Reference (URL)
2021	NCT04699656	Phase 1	Plazomicin Study in ESRD Patients Receiving IHD	https://ClinicalTrials.gov/show/NCT04699656 (accessed on 27 October 2022)
2017	NCT03270553	Phase 1	A Study to Assess the Effect of Plazomicin on the Pharmacokinetics of Metformin	https://ClinicalTrials.gov/show/NCT03270553 (accessed on 27 October 2022)
2017	NCT03177278	Phase 1	A Study to Assess the Metabolism, Excretion, and Mass Balance of Radio-Labeled Plazomicin	https://ClinicalTrials.gov/show/NCT03177278 (accessed on 27 October 2022)
2012	NCT01462136 Published in 2018	Phase 1	PK Study of ACHN-490 Injection in Renally Impaired Subjects	https://ClinicalTrials.gov/show/NCT01462136 (accessed on 27 October 2022)
2012	NCT01514929 Published in 2019	Phase 1	A Study to Evaluate the Effect of IV ACHN-490 Injection on the QT/QTc Interval in Healthy Volunteers	https://ClinicalTrials.gov/show/NCT01462136 (accessed on 27 October 2022)
2010	NCT01034774	Phase 1	Phase 1 Study to Determine Safety, Blood PK and Lung Penetration	https://ClinicalTrials.gov/show/NCT01034774 (accessed on 27 October 2022)
2009	NCT00822978 Published in 2012	Phase 1	Phase 1 Study for Safety of ACHN-490	https://ClinicalTrials.gov/show/NCT00822978 (accessed on 27 October 2022)
2018	NCT01096849	Phase 2	A Multicenter, Randomized, Double-Blind, Phase 2 Study of the Efficacy and Safety of Plazomicin Compared with Levofloxacin in the Treatment of Complicated Urinary Tract Infection and Acute Pyelonephritis	https://clinicaltrials.gov/ct2/show/NCT01096849 (accessed on 27 October 2022)
2012	NCT01096849	Phase 2	A Study of Plazomicin Compared with Levofloxacin for the Treatment of Complicated Urinary Tract Infection (cUTI) and Acute Pyelonephritis (AP)	https://ClinicalTrials.gov/show/NCT01096849 (accessed on 27 October 2022)
2019	NCT01970371 Published in 2018 and 2019	Phase 3	Evaluation of Plazomicin, Tigecycline, and Meropenem Pharmacodynamic Exposure against Carbapenem-Resistant Enterobacteriaceae in Patients with Bloodstream Infection or Hospital-Acquired/Ventilator-Associated Pneumonia from the CARE Study (ACHN-490-007)	https://ClinicalTrials.gov/show/NCT01970371 (accessed on 27 October 2022)
2019	NCT02486627	Phase 3	Once-Daily Plazomicin for Complicated Urinary Tract Infections	https://ClinicalTrials.gov/show/NCT02486627 (accessed on 27 October 2022)
2017	NCT00676169 Published in 2018 and 2019	Phase 3	Microbiological outcomes with plazomicin (PLZ) versus meropenem (MEM) in patients with complicated urinary tract infections (CUTI), including acute pyelonephritis (AP) in the epic study	https://ClinicalTrials.gov/show/ NCT00676169 (accessed on 27 October 2022)
2016	NCT01970371	Phase 3	A Study of Plazomicin Compared with Colistin in Patients with Infection Due to Carbapenem-Resistant Enterobacteriaceae (CRE)	https://ClinicalTrials.gov/show/ NCT01970371 (accessed on 27 October 2022)
2016	2015-001588-37	Phase 3	A Phase 3, Randomized, Multicenter, Double-Blind Study to Evaluate the Efficacy and Safety of Plazomicin Compared with Meropenem followed by Optional Oral Therapy for the Treatment of complicated urinary tract infections	https://www.clinicaltrialsregister.eu/ctr-search/search?query=eudract_number:2015-001588-37 (accessed on 27 October 2022)
2016	2013-001997-18	Phase 3	A Phase 3, Multicenter, Randomized, Open-Label Study to Evaluate the Efficacy and Safety of Plazomicin Compared with Colistin in Patients with Infection due to Carbapenem-Resistant Enterobacteriaceae (CRE)	https://www.clinicaltrialsregister.eu/ctr-search/trial/2013-001997-18/results (accessed on 27 October 2022)

Synthesis of the published RCTs on the use of plazomicin in clinical practice. The table describes the year of publication, the identification number of each trial, the title, and the URL where the trial is shown extensively.

**Table 4:** Synthesis of the comparators detected in the included RCTs.

Antibiotic Used As Comparator	Number of Studies	Percentage
Meropenem	7	43.75%
Colistin	5	31.25%
Levofloxacin	1	6.25%
Ceftazidime-Avibactam	2	12.50%
Meropenem-Vaborbactam	1	6.25%
Cefiredocol	1	6.25%
Ceftolozane-Tazobactam	1	6.25%
Tigecycline	1	6.25%

Description of the antibiotics used as comparators of plazomicin in the treatment of complicated infections by multi-resistant pathogens. In the table, the number of studies in which the antimicrobial comparator is used is presented (the number of studies is also expressed as a percentage).

**Table 5:** Bacteria species detected in studies conducted on clinical isolates.

Bacteria Species	Number of Studies	Number of Studies (%)
Multiple bacteria *	3	4.55%
*Staphylococcus aureus*	3	4.55%
*Pseudomonas aeruginosa*	3	4.55%
*Klebsiella Pneumoniae*	16	24.24%
*Gram-negative* spp. **	6	9.09%
*Enterobacterales* spp.	12	18.18%
*Enterobacteriaceae* spp.	19	28.79%
ENT ***	12 + 19	46.97%
*Escherichia coli*	6	9.09%
*Acinetobacter baumannii*	3	4.55%
*Brucella* spp.	1	1.52%
*Enterococcus Faecium*	1	1.52%
Gram-positive spp. **	1	1.52%
*Enterobacter* spp.	1	1.52%

Description of the bacteria isolated from 66 studies on clinical isolates tested with plazomicin and included in the review. Notes: * Bacteria isolated by screening samples; ** data not available; *** *Enterobacterales* summed to *Enterobacteriaceae*.

**Table 6:** Antibiotic-resistant strains detected among the studies on isolates.

Type of Antibiotic Resistance	Number of Studies	Number of Studies (%)
Carbapenem-resistant (none declared specifically)	23	43.40%
MDR	15	28.30%
NDM	2	3.77%
ESBLs	4	7.55%
AME	3	5.66%
KPC	6	11.32%
ESCREC	1	1.89%
Quinolone-resistant	1	1.89%
MRSA	3	5.66%

Description of the antibiotic resistance found in 53/66 studies on isolates. Notes: MDR, multidrug-resistant; NDM, New Delhi metallo-beta-lactamase; ESBLs, extended spectrum beta-lactamases; AME, aminoglycoside-modifying enzyme; KPC, *K. pneumoniae* carbapenemase; ESCREC, extended-spectrum cephalosporin-resistant *E. coli*; MRSA, methicillin-resistant *S. aureus.*

## Data Availability

Not applicable.

## Appendix A

**Table A1:** The Search Strings Used for Each Database and the Relative Number of Studies Detected.

Database	Search String	Studies Detected (Number)
MEDLINE (via Ovid)	plazomicin.mp. achn 490.mp. zemdri.mp. 4. 1 or 2 or 3	192
EMBASE	‘plazomicin’/exp OR ‘achn 490’ OR ‘achn490’ OR ‘plazomicin’ OR ‘plazomicin sulfate’ OR ‘plazomicin sulphate’ OR ‘zemdri’	504
CENTRAL	“plazomicin” OR “achn 490” OR “zemdri”	23
ClinicalTrials.gov	plazomicin OR achn490 OR zemdri	10
EU-CTR	plazomicin OR achn490 OR zemdri	2
